# Activation of TGF‐*β* activated kinase 1 promotes colon mucosal pathogenesis in inflammatory bowel disease

**DOI:** 10.14814/phy2.13181

**Published:** 2017-04-03

**Authors:** Zhiwei Liu, Fansheng Kong, Jefferson E. Vallance, Eleana Harmel‐Laws, Surya Amarachintha, Kris A. Steinbrecher, Michael J. Rosen, Sandip Bhattacharyya

**Affiliations:** ^1^ Department of PediatricsCenter for Prevention of Preterm BirthCincinnati Children's Hospital Medical CenterUniversity of CincinnatiCincinnatiOhio; ^2^ Division of Gastroenterology, Hepatology and NutritionDepartment of PediatricsCincinnati Children's Hospital Medical CenterUniversity of CincinnatiCollege of MedicineCincinnatiOhio; ^3^ Present address: Neonatal DivisionInternational Peace Maternity and Child Health HospitalSchool of MedicineShanghai Jiao Tong UniversityMinhang QuChina

**Keywords:** Glucocorticoid, intestinal inflammation, TAK1

## Abstract

The etiology and mechanisms for inflammatory bowel disease (IBD) are incompletely known. Determination of new, clinically important mechanisms for intestinal inflammation is imperative for developing effective therapies to treat IBD. We sought to define a widespread mechanism for colon mucosal inflammation via the activation of TGF‐*β* activated Kinase 1 (TAK1), a central regulator of cellular inflammatory actions. Activation of TAK1 and the downstream inflammatory signaling mediators was determined in pediatric patients with ulcerative colitis (UC) or Crohn's disease (CD) as well as in DSS‐induced and spontaneous IBD in mice. The role of TAK1 in facilitating intestinal inflammation in murine models of IBD was investigated by using (5Z)‐7‐Oxozeaenol, a highly selective pharmacological inhibitor of TAK1. We found hyper‐activation of TAK1 in patients with UC or CD and in murine models of IBD. Pharmacological inhibition of TAK1 prevented loss in body weight, disease activity, microscopic histopathology, infiltration of inflammatory cells in the colon mucosa, and elevated proinflammatory cytokine production in two murine models of IBD. We demonstrated that at the early phase of the disease activation of TAK1 is restricted in the epithelial cells. However, at a more advanced stage of the disease, TAK1 activation predominantly occurs in nonepithelial cells, especially in macrophages. These findings elucidate the activation of TAK1 as crucial in promoting intestinal inflammation. Thus, the TAK1 activation pathway may represent a suitable target to design new therapies for treating IBD in humans.

## Introduction

Patients suffering from IBD exhibit chronic intestinal inflammation due to dysregulated mucosal immune response and epithelial barrier disruption (Xavier and Podolsky 2007). Despite many years of extensive research indicating a complex interplay of genetic, microbial, and environmental contributions as possible factors associated with aberrant intestinal inflammation, the etiology and mechanisms for disease pathogenesis remain elusive (Xavier and Podolsky 2007). Hence, currently available therapies for pediatric IBD frequently fail to resolve inflammation (Kozuch and Hanauer 2008). For example, 50% of the patients that undergo glucocorticoid (GC) therapy develop steroid‐dependency or require surgery (Aceituno et al. 2008); 20–30% of patients are GC nonresponders (Rosen et al. 2015). Anti‐TNF therapy, so far the best treatment for moderate‐to‐severe IBD, promotes mucosal healing in only 30–50% of the patients (Colombel et al. 2010, 2011). Defining new, clinically relevant mechanisms for intestinal inflammation is a clear need for developing efficient therapies in IBD.

Targeting of signaling mediators of inflammatory pathways, such as MAP kinases (MAPKs) and JAK‐STAT is an emerging field in developing therapies in IBD (Van Den Blink et al. 2002; Bamias et al. 2016). Activation of TGF‐*β* activated Kinase 1 (TAK1) is crucial in engaging multiple innate immune signaling pathways including NF*κ*B, Activated Protein1 (AP1), MAPK, and STAT that promote inflammatory responses in IBD (Kawai and Akira 2006; Abbott et al. 2007; Adhikari et al. 2007). TAK1 is a serine/threonine kinase in the MAPK kinase kinase (MAP3K) family and is induced by a spectrum of stimuli (Sakurai 2012). Involvement of TAK1 in inflammatory ailments is well demonstrated (Ma et al. 2011; Neubert et al. 2011; Ridder et al. 2011; Ridder and Schwaninger 2012; Sakurai 2012). Activation of TAK1 promotes clinical complications in the digestive system. TAK1 is activated in colon cancer (Takahashi et al. 2015), and inhibition of TAK1 reduced colon tumor cell growth (Singh et al. 2012). Also, blocking TAK1 activation decreases chemoresistance in pancreatic cancer (Melisi et al. 2011).

The role of TAK1 in IBD is not clear. Deletion of TAK1 in T‐cell–induced intestinal inflammation (Sato et al. 2006). The same group later found that in these mice TAK1 expression is retained in CD4^+^ T‐cells. These cells acquire an inflammatory phenotype and continuous access to colon that result in pathogenesis in colitis (Sanjo et al. 2015). Also, TAK1 in epithelial cells was protective in mouse colitis (Kajino‐Sakamoto et al. 2008; Kim et al. 2009). Enterocyte‐specific constitutive deletion of TAK1 in mice developed spontaneous intestinal inflammation (Kajino‐Sakamoto et al. 2008). TAK1 in intestinal epithelial cells exhibited cytoprotective roles in chemical (DSS)‐induced colitis (Kim et al. 2009). The protective role of TAK1 is believed to be due to the maintenance of Paneth cell integrity via restraining necroptosis (Simmons et al. 2016). In contrast, phosphorylation/activation of TAK1 was increased in ileum from patients with CD and correlated with tissue fibrosis (Grillo et al. 2015). Also, TAK1 was activated in TNBS‐induced colitis (Lim et al. 2015). Deletion of CYLD, a TAK1 deubiquitinating enzyme in T‐cells resulted in TAK1 hyper‐activation and spontaneous intestinal inflammation (Reiley et al. 2007). Raf kinase inhibitor protein (RKIP) promoted intestinal epithelial cell apoptosis in human and mouse IBD via interaction with TAK1 (Lin et al. 2016). Also, a recent GWAS study suggested association of TAB 2, a TAK1‐binding protein with IBD (Liu et al. 2015). However, the functional role of TAK1 in mediating mucosal pathogenesis in IBD remains to be determined.

Here, we test the hypothesis that activation of TAK1 mediates intestinal inflammation and pathogenesis in IBD. We provide evidence for hyper‐activation of TAK1 in pediatric patients with UC and CD, as well as in chemically induced and spontaneous murine IBD. We demonstrate that GC, a mainstay therapy for IBD, fails to suppress the activation of TAK1 and downstream signaling mediators that induce intestinal inflammation. Our comprehensive analyses, utilizing TAK1‐specific pharmacological inhibition, provide a direct evidence for TAK1 involvement in promoting mucosal pathogenesis in two different mouse models of IBD. These results implicate activation of TAK1 as an important mechanism for intestinal inflammation. Our studies suggest that ineffective suppression of TAK1 activation may be responsible for the limited efficacy of the interventions presently available for treating IBD.

## Materials and Methods

### Materials

TAK1 inhibitors (5Z)‐7‐Oxozeaenol (EMD Millipore, Billerica, MA), Dexamethasone, Piroxicam (Sigma, St. Louis, MO) were purchased and reconstituted as per the manufacturer's instructions. Antibodies used in this study were as follows: anti‐actin (A 5060) (Sigma); anti‐phospho‐TAK1 (06‐1425) (Millipore, Temecula, CA); anti‐phospho‐TAK1 (109404) (Abcam, Cambridge, MA); anti‐TAK1 (5206, 4505), anti‐phospho‐SAPK/JNK (4668, 9251), anti‐SAPK/JNK (9253, 9252), anti‐phospho‐p38 MAPK (4511, 9211), anti‐p38 MAPK (9211, 9212), anti‐phospho‐I*κ*B*α* (2859) and anti‐I*κ*B*α* (4814, 9242) (Cell Signaling Technology, Beverly, MA); anti‐TAK1 (7162) (Santa Cruz Biotechnology, Santa Cruz, CA).

### Mice

Male and female mice (Jackson Laboratories, Bar Harbor, ME) were group‐housed at Cincinnati Children's Hospital Medical Center (CCHMC) Animal Facility under controlled temperature (25°C) and photoperiods (12 h light and 12 h dark cycle). Mice used for the experimentation were of C57BL/6 background, 6–8 weeks old (18–22 g) and allowed unrestricted access to standard diet and tap water. For each group of experiments, mice were matched by age, sex, and body weight.

### Human samples

Rectosigmoid biopsies were obtained from the Division of Gastroenterology, Cincinnati Children's Hospital Medical Center (CCHMC). Male and female pediatric patients (ages 4–18 years) with CD or UC, and patients found not to have intestinal inflammation (non‐IBD) were recruited under approved IRB protocol. Patients undergoing colonoscopy for other routine clinical indications were enrolled to obtain non‐IBD controls. Patients with indeterminate IBD were excluded from these studies. Prospectively collected endoscopic biopsy tissues were snap frozen at endoscopy and stored at −80°C.

### DSS model of colitis

Acute colitis was induced by feeding male and female mice with 3% DSS (wt/vol) (mol wt 36–50 kDa; MP Biomedicals, Solon, OH) dissolved in drinking water. Fresh DSS solution was prepared daily and fed ad libitum for 7 days (Okayasu et al. 1990). The bedding was changed weekly and dirty cage bedding was transferred between cages to ensure a homogenous microbial environment. For the recovery model of colitis, mice received 3% DSS for 5 days followed by unsupplemented normal drinking water for 2 weeks.

### IL‐10^−/−^ model of IBD

In the preliminary studies, phosphorylation of TAK1 was assessed in IL‐10^−/−^ male and female mice (10–12 weeks old, C57BL/6 background). At this age, IBD was developed spontaneously (Kawachi et al. 2000). To synchronize the onset and accelerate the development of IBD, IL‐10^−/−^ male and female mice (6–8 weeks old) were administered 200 ppm piroxicam (NSAID) in chow for 9 days (Holgersen et al. 2014).

### In vivo treatment with TAK1 inhibitor

TAK1 inhibitor (5Z)‐7‐Oxozeaenol (Oxo) was resuspended as a 10 mg/mL stock in DMSO. This was further diluted in peanut oil (Sigma) to yield a lower concentration of the drug stock in 10% DMSO (Kong et al. 2015). Mice were injected with various doses of (5Z)‐7‐Oxozeaenol intraperitoneally. Alternatively, 10% DMSO in peanut oil was delivered as a vehicle control.

### Tissue and plasma sampling

Colonic tissue and plasma samples were collected at time points as illustrated in the relevant figures. At each time point, blood was collected by retro‐orbital phlebotomy into heparinized capillary tubes, with the time from first handling the animal to completion of the bleeding not exceeding 30 sec. Blood was collected in tubes containing EDTA, and plasma was frozen and kept at −80°C. The intestines were excised and rinsed with ice‐cold PBS. The colon was cut close to the ileocecal valve and rectum, and the length was measured. Sections (1 cm) of the distal and proximal colon were cut out and opened longitudinally, fixed in 4% formalin solution, and embedded in paraffin for histological analysis. The remaining part of the colon was weighed and frozen in liquid nitrogen for subsequent molecular analysis.

### Evaluation of disease activity

Mice were observed twice daily for water/food consumption, weight, morbidity, stool consistency, and the presence of blood in feces and at the anus. The disease activity index (DAI) was calculated by assigning well‐established and validated scores for parameters (Cooper et al. 1993).

### Evaluation of histopathology

4 *μ*m thick paraffin‐embedded sections were subjected to H&E staining for histological analysis. Histological scores were blindly determined as per Obermeier et al. (1999).

### Immunofluorescence

For immunofluorescence staining of phosphor‐TAK1, 2 *μ*m frozen colon sections were used. Sections were stained with rabbit monoclonal anti‐TAK1 (ab109404; Abcam) followed by goat anti‐rabbit‐Alexa Fluor‐488 (ab150077; Abcam). For the nuclear counter staining, sections were stained with DAPI (Life Technologies).

### Radioimmunoassay

Plasma concentrations of corticosterone were determined by radioimmunoassay from blood collected by retro‐orbital phlebotomy as described previously (Boyle et al. 2005). The corticosterone assays were performed as per the manufacturer's instructions (MP Biomedicals, Orangeburg, NY).

### Cytokine measurement

In the DSS model of IBD, colons from the colocecal junction to the anus were excised at the indicated time points. Preliminary experiments indicated higher levels of proinflammatory cytokines in the distal colon than other parts of colon. These findings were concordant with more severe histological changes in the distal colon compared with the other parts of colon (data not shown). Based on these results, the distal colon was selected for cytokine measurement. The distal colon (2 cm from the rectum) was excised and snap‐frozen. Samples were thawed at room temperature, homogenized in ice‐cold PBS containing protease inhibitor cocktails (Sigma). Tissue processing was performed with TissueLyserII (Qiagen, Valencia, CA). Tissue homogenates were centrifuged at 3000*g* for 10 min at 4°C. The supernatants were separated into aliquots and frozen at −80°C until analyses. In the IL‐10^−/−^ model of IBD, the whole colonic tissues were homogenized due to heterogeneous pathogenesis throughout the colon in this model. Concentrations of TNF‐*α*, IL‐6, and IL‐12 in tissue homogenates samples were measured by enzyme‐linked immunosorbent assay (ELISA) as per the manufacturer's instructions (BD Biosciences PharMingen, San Diego, CA). Cytokine concentrations are expressed as picograms per 100 *μ*g of colonic tissue.

### Immunoblot analysis

Colon tissues were disrupted with TissueLyserII in lysis buffer (20 mmol/L Tris‐HCl pH 7.5, 150 mmol/L NaCl, 2 mmol/L EDTA, 1% NP‐40) containing protease and phosphatase inhibitor cocktails, and supernatants were collected. Samples were resolved as described previously (Bhattacharyya et al. 2010). Blots were stripped with Restore^TM^ Western Blot Stripping Buffer (Thermo Scientific, Rockford, IL) and reprobed with different antibodies. Band intensities were quantified by densitometry analyses (Image J, NIH).

### Statistical analysis

All experiments were replicated three times to assure the reproducibility of the observations. Data are expressed as the mean ± SEM. For experiments yielding continuous data in three or more groups, ANOVA was applied as a global test for differences in the primary outcome variable. Histopathological scoring, as an ordinal variable, was compared among groups using the nonparametric Kruskal–Wallis test. Pair‐wise comparisons were made only when an overall effect was detected using the Student's *t* test or Mann–Whitney *U* test followed by Hochberg procedure to adjust for multiple comparisons. Repeated‐measures two‐way ANOVA with Bonferroni correction was used to compare measurements of mouse weight over time among experimental groups. Statistical analyses were performed using Prism 6.0 h (GraphPad Software, La Jolla, CA).

### Ethical considerations

All authors concur with the submission of the manuscript. The current work has not been submitted or published elsewhere. Pediatric patients were enrolled through protocols approved by the Institutional Review Boards at CCHMC (IRB 2013‐4749). Animal studies were performed following recommendations in the Guide for the Care and Use of Laboratory Animals of the National Institutes of Health. The experimental protocols were approved by the Animal Care and Use Committee of CCHMC.

## Results

### Activation of TAK1 and the downstream signaling mediators in pediatric IBD

**Figure 1:**
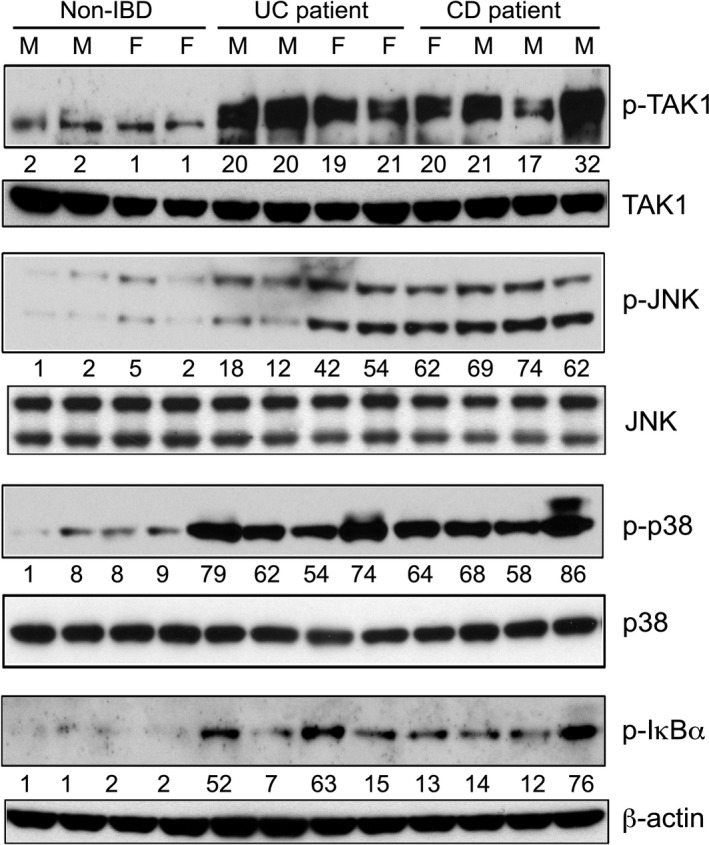
Phosphorylation of TAK1 and the downstream signaling mediators in pediatric IBD. Rectal biopsies from non‐IBD, male (M) and female (F) patients were harvested. The sample size for each group: *n* = 8. Tissue lysates were analyzed by immunoblot analysis using anti‐phospho TAK1 (p‐TAK1), total TAK1 (TAK1); anti‐phospho JNK (p‐JNK), total JNK (JNK); anti‐phospho p38 MAPK (p‐p38), total p38 MAPK (p38); anti‐phospho I*κ*B*α* and *β*‐actin antibodies. Western blots were quantified by densitometric analyses (Image J, NIH). The lowest abundance of phospho‐proteins in non‐IBD patient is considered as onefold. Abundance of phospho‐proteins were normalized to total protein or *β*‐actin, as appropriate.

Initially, we examined whether TAK1 is activated in pediatric patients with active UC or CD. To address this, study subjects with moderate to severe UC or CD were recruited. Phosphorylation of TAK1 is considered as a hallmark for its activation. We assessed TAK1 phosphorylation in the rectal biopsy tissues from male and female patients with or without IBD. Patients found not to have intestinal inflammation (non‐IBD) were considered as the control for these studies. Low levels of TAK1 phosphorylation were observed in most (7/8) of the non‐IBD patients (Table 1). However, biopsies from UC (7/8) and CD (6/8) patients exhibited high levels of TAK1 phosphorylation (Table 1). Densitometric analysis of the immunoblots indicated 8.6 (*P* = 0.001) and 9.8 (*P* = 0.005)‐fold increase in TAK1 phosphorylation in patients with UC and CD, respectively, compared to non‐IBD patients (Fig. 1). Activity of TAK1 stimulates the downstream signaling mediators and transcription factors, such as JNK, p38 MAPK, and NF*κ*B. Consistent with TAK1 activation, phosphorylation of JNK was upregulated in patients with IBD; more prominently in patients with CD (Fig. 1). All non‐IBD patients (8/8) exhibited low levels of JNK phosphorylation. In contrast, all CD (8/8) and most of the UC (6/8) patients demonstrated high levels of JNK phosphorylation (Table 1). Also, all UC (8/8) and CD (8/8) patients revealed high‐p38 MAPK phosphorylation (Fig. 1). Phosphorylation of I*κ*B*α* is required for the recruitment and activation of NF*κ*B. In non‐IBD patients, I*κ*B*α* phosphorylation was found to be very low (Fig. 1). Most of the UC (5/8) and CD (6/8) patients exhibited moderate I*κ*B*α* phosphorylation (Table 1). Together, TAK1 as well as the signaling intermediates downstream to TAK1 that mediate inflammatory reactions were markedly activated in male and female patients with UC or CD.

**Table 1:** Expression of phosphorylated signaling proteins in rectal biopsy specimens

Groups	Patient No.	p‐TAK1 expression		p‐Tκβα expression		p‐JKN expression		p‐p38 expression	
		Low (%)	High (%)	Low (%)	High (%)	Low (%)	High (%)	Low (%)	High (%)
Healthy control	8	7 (88)	1 (12)	8 (100)	0 (0)	8 (100)	0 (0)	6 (75)	2 (25)
UC	8	1 (22)	7 (88)	3 (36)	5 (64)	2 (25)	6 (75)	0 (0)	8 (100)
CD	8	2 (25)	6 (75)	2 (25)	6 (75)	0 (0)	8 (100)	0 (0)	8 (100)

Expression of phospho‐TAK1, phospho‐IκBα, phospho‐JNK, phospho‐p38MAPK were analyzed by immunoblot analysis. The low‐ and high‐grade phosphorylation of the signaling proteins was determined empirically. The percentage within parentheses indicates the proportion of patients with high or low levels of the relevant phosphorylated protein.

### Activation of TAK1 and the downstream signaling mediators in mouse models of IBD

**Figure 2:**
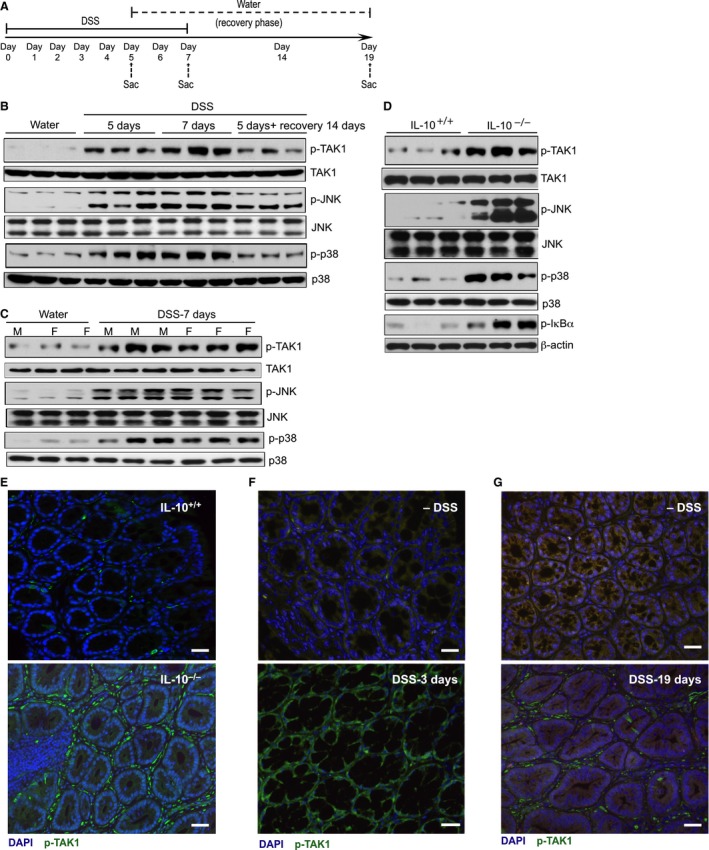
Phosphorylation of TAK1 and the downstream signaling mediators in murine models of IBD. (A) Treatment paradigm for DSS‐induced IBD. In acute model of IBD, male C57BL/6 mice (*n* = 8–12/group) received 3% DSS or tap water, and sacrificed as indicated. In semichronic model of IBD, male C57BL/6 mice (*n* = 8–10/group) received 3% DSS or tap water for 5 days and thereafter only tap water for another 14 days and sacrificed as indicated. (B) Colons were harvested and tissue homogenates were analyzed by immunoblot analysis. (C) Male and female C57BL/6 mice (*n* = 8–10/group) received 3% DSS or tap water for 7 days, and sacrificed on day 7. Colons were harvested and tissue homogenates were analyzed by immunoblot analysis. (D) Colons from 10 to 12 weeks old IL‐10^+/+^ and IL‐10^−/−^ mice (*n* = 6/group) were harvested. Tissue homogenates were analyzed by immunoblot analysis using anti‐phospho TAK1 (p‐TAK1), total TAK1 (TAK1); anti‐phospho JNK (p‐JNK), total JNK (JNK); anti‐phospho p38 MAPK (p‐p38), total p38 MAPK (p38); anti‐phospho I*κ*B*α* and *β*‐actin antibodies. (E) Representative immunofluorescence images show increased phosphorylated TAK1^+^ (green) cells in IL‐10^−/−^ colons compared to IL‐10^+/+^ colons. (F) Representative immunofluorescence images show increased phosphorylated TAK1^+^ (green) cells in DSS treated colons compared to colons without DSS treatment. Magnification: 100×; scale bar: 100 *μ*mol/L.

To understand the role of TAK1 in mediating the pathogenesis in IBD, DSS‐induced colitis in wild‐type C57BL/6 mice and spontaneous IBD in IL‐10^−/−^ mice (C57BL/6 background) were used as surrogates for human IBD. Mice were treated with 3% DSS to induce acute colitis as demonstrated in Figure 2A. The semichronic model of DSS‐colitis is validated by several independent studies (Melgar et al. 2005; Sha et al. 2013). In the absence of DSS, phosphorylation of TAK1, JNK, and p38 MAPK in the colonic tissues were either undetectable or very low (Fig. 2B). However, substantial increases in TAK1, JNK, and p38 MAPK phosphorylation were observed after DSS treatment for 5 days. Consequences of DSS treatment on the phosphorylation of inflammatory signaling mediators were more pronounced after 7 days of treatment, the time point known to have more severe intestinal inflammation. In the recovery/semichronic phase of the disease, phosphorylation of TAK1, JNK, and p38 MAPK were reduced, but still higher than control mice, which were not treated with DSS (Fig. 2B). The above experiments were performed with male mice. To examine whether the activation of TAK1 and downstream signaling mediators in DSS‐induced colitis is sex‐dependent, similar studies were performed with age‐matched male and female mice. There was no significant difference in phosphorylation of TAK1, JNK, and p38 MAPK between male and female mice (Fig. 2C).

The experiments with IL‐10^−/−^ mice were performed with adult mice (10–12 weeks old) to ensure detectable intestinal inflammation. Phosphorylation of TAK1, JNK, p38, and I*κ*B*α* was elevated in IL‐10^−/−^ mice compared to control IL‐10^+/+^ mice (Fig. 2D). These results were further supported by immunoflorescence analyses indicating substantial increase in phospho‐TAK1^+^ cells in IL‐10^−/−^ mice compared to IL‐10^+/+^ mice (Fig. 2E). The majority of phospho‐TAK1^+^ cells were nonepithelial cells. At the early phase of DSS‐colitis, after 3 days of DSS treatment a marked enhancement of phospho‐TAK1 abundance was observed predominantly in the epithelial cells (Fig. 2F). At this phase of the disease, minimal epithelial damage was detected. In contrast, in the recovery phase of DSS‐colitis, on day 19 phospho‐TAK1 in nonepithelial cells was markedly elevated compared to control mice, those are not treated with DSS (Fig. 2G). This increased nonepithelial TAK1 expression was also found in the colons from IL‐10^−/−^ mice (Fig. 2E). Together, consistent with the data obtained from IBD patients the activation TAK1 and its downstream signaling mediators were substantially upregulated in two different murine models of IBD.

### Pharmacological inhibition of TAK1 reduces pathogenesis in DSS‐induced acute IBD

**Figure 3:**
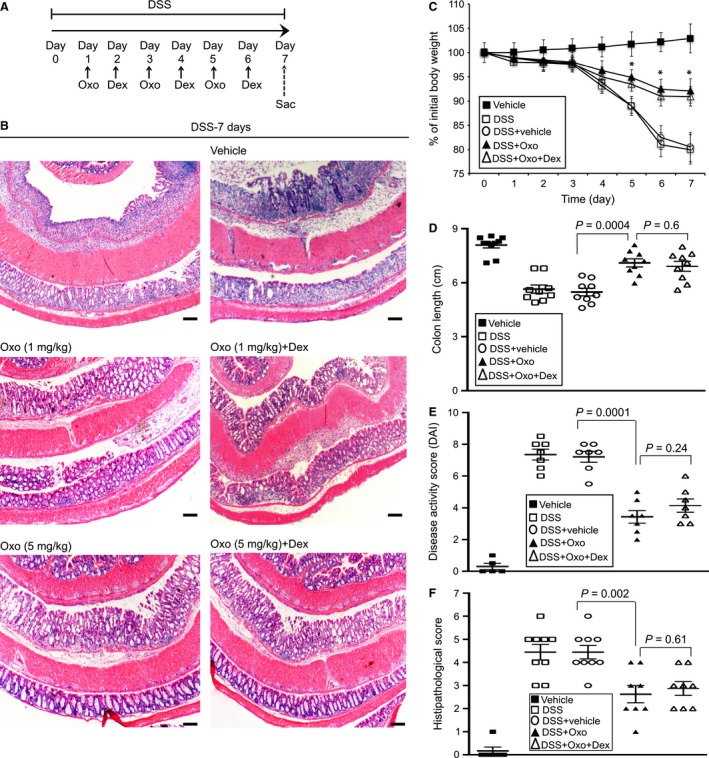
Pharmacological inhibition of TAK1 reduces pathogenesis in DSS‐induced acute IBD. (A) Male and female C57BL/6 mice (*n* = 12–15 per group) were on 3% DSS. Mice were treated with Dex (1 mg/kg body weight) and TAK1 inhibitor Oxo or vehicle (DMSO + mineral oil) (1 or 5 mg/kg) and sacrificed as indicated. (B) H&E stained colon tissues. Magnification: 200×; scale bar: 100 *μ*mol/L. (C) Loss in body weight. Data presented as mean ± SEM. *, *P* < 0.05 versus DSS+vehicle. (D) Colon lengths, (E) Disease activity and (F) Histopathology were determined. All data presented as mean ± SEM.

To examine whether inhibition of TAK1 activation rescues DSS‐mediated pathogenesis, mice were treated with (5Z)‐7‐Oxozeanol (Oxo), a highly selective pharmacological inhibitor of TAK1 widely used for preclinical studies (Ninomiya‐Tsuji et al. 2003; Winssinger and Barluenga 2007; Neubert et al. 2011; Singh et al. 2012). Another group of mice were treated with both Oxo and Dex to assess whether the combination therapy provide beneficial effects. As TAK1 contributes to both immune and nonimmune cellular functions, we first determined the optimal Oxo dose that does not incur overt toxicity. For these studies, mice exposed to DSS were treated with various doses of Oxo (1, 5, 10 mg/kg body weight). Plasma ALT, GOT, AST, and creatinine levels were examined. Toxicity from Oxo treatment was only observed at and higher than a dose of 10 mg/kg body weight (data not shown). Subsequent experiments with Oxo were conducted at doses of 1 and 5 mg/kg body weight. Mice were treated with Oxo and sacrificed as illustrated in Figure 3A. Our initial studies were focused on acute IBD, while mice were on DSS for 7 days. There was no detectable effect of treatment with vehicle (DMSO + mineral oil) on DSS‐induced epithelial degeneration and infiltration of inflammatory cells in the colon mucosa (Fig. 3B). However, treatment with Oxo reduced loss in crypts, epithelial damage, and the infiltration of inflammatory cells in the colon mucosa (Fig. 3B). GC treatment is a mainstay therapy for the acute flares of IBD. We examined whether treatment with dexamethasone (Dex, a synthetic GC) results in additional benefits in Oxo‐treated DSS‐exposed mice. There was no significant difference in epithelial architecture or immune cell infiltration between Oxo and Oxo+Dex–treated groups. Oxo treatment significantly prevented the loss in body weight (*P* < 0.05; Fig. 3C) and colon length shortening (*P* = 0.0004; Fig. 3D) in DSS‐exposed mice. Again, treatment with Dex had no additional beneficial effect on above parameters of IBD pathogenesis. The DAI in DSS‐exposed mice was reduced by 59% in Oxo (5 mg/kg body weight)‐treated mice compared to vehicle‐treated mice (Fig. 3E; *P* = 0.0001). Also, treatment with Oxo prevented histopathological score by 61% (Fig. 3F; *P* = 0.002) in comparison with the vehicle‐treated group. Once again, there was no significant difference between Oxo and Oxo+Dex–treated groups.

**Figure 4:**
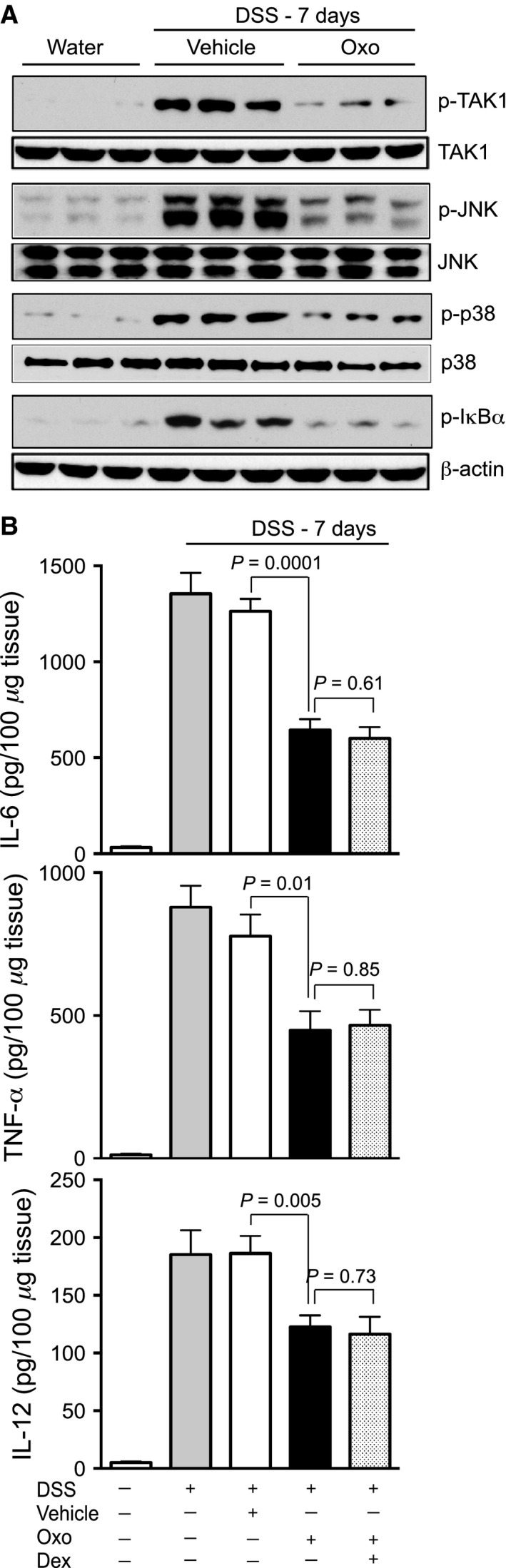
Pharmacological inhibition of TAK1 prevents expression of inflammatory signaling mediators and cytokine in acute IBD. Male and female C57BL/6 mice (*n* = 12–15 per group) were on 3% DSS for 7 days. Mice were treated with TAK1 inhibitor Oxo or vehicle (DMSO + mineral oil) (5 mg/kg) and sacrificed as illustrated in (A). (A) Colon tissue homogenates were analyzed by immunoblot analysis using anti‐phospho TAK1 (p‐TAK1), total TAK1 (TAK1); anti‐phospho JNK (p‐JNK), total JNK (JNK); anti‐phospho p38 MAPK (p‐p38), total p38 MAPK (p38); anti‐phospho I*κ*B*α* and *β*‐actin antibodies. (B) Expression of IL‐6, TNF‐*α* and IL‐12 in colon homogenates were analyzed by ELISA. Data presented as mean ± SEM.

The efficacy of Oxo in restricting activation of inflammation‐inducing signaling mediators was determined. Substantial increases in the phosphorylation of TAK1, JNK, p38 MAPK, and I*κ*B*α* in the colonic tissues were observed in vehicle‐treated mice exposed to DSS (Fig. 4A). However, treatment with Oxo markedly reduced phosphorylation of all signaling intermediators investigated in these studies (Fig. 4A). Since proinflammatory cytokines promote intestinal inflammation, effects of TAK1 inhibition on cytokine synthesis were evaluated. Elevated expression of IL‐6, TNF‐*α*, and IL‐12 were not affected by the treatment with vehicle (Fig. 4B). However, treatment with Oxo significantly inhibited expression of IL‐6 (*P* = 0.0001), TNF‐*α* (*P* = 0.01), and IL‐12 (*P* = 0.005) induced by DSS exposure (Fig. 4B).

### Inhibition of TAK1 enhances recovery from DSS‐induced colitis in mouse

**Figure 5:**
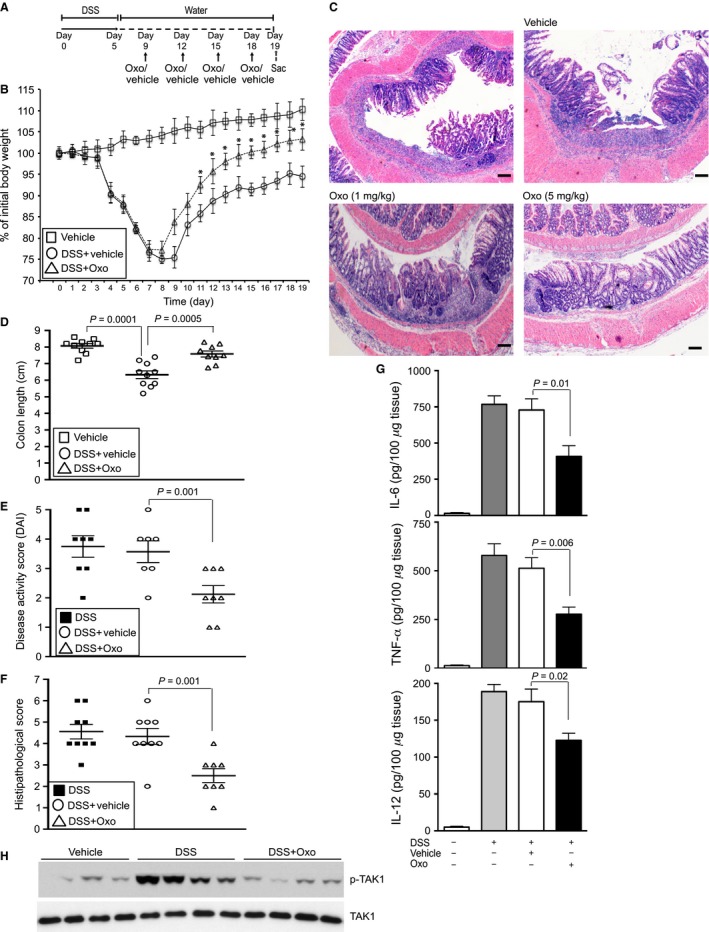
Inhibition of TAK1 reduces pathogenesis in the recovery phase of DSS‐induced IBD. (A) Male and female C57BL/6 mice (*n* = 12–15 per group) were on 3% DSS and with vehicle (DMSO+mineral oil) or Oxo (1 or 5 mg/kg body weight) and sacrificed as indicated. (B) Loss in body weight. Data presented as mean ± SEM. *P < 0.05 versus DSS+vehicle. (C) H&E stained colons tissues; magnification: 200×; scale bar: 100 *μ*mol/L. (D) Colon lengths, (E) Disease activity and (F) Histopathology were determined. (B) Expression of IL‐6, TNF‐*α* and IL‐12 in colon homogenates were analyzed by ELISA. Data presented as mean ± SEM.

Next, we assessed the effect of TAK1 inhibition in recovery from DSS‐induced injury and inflammation. Mice were treated and sacrificed as depicted in Fig. 5A. To evaluate the effect of 5 days of DSS exposure on the recovery phase of IBD, mice were monitored for an additional 14 days. The acute pathogenesis was not resolved after the removal of DSS. Vehicle‐treated mice continued to lose body weight even after removal of DSS until day 11, while they started to regain the weight (Fig. 5B). There was no detectable rectal bleeding at days 10–19; however, the disease activity (including diarrhea) and histopathology score remained high (Fig. 5 E and F). The regaining of body weight improved in Oxo‐treated mice (Fig. 5B). Significant regaining in body weight in Oxo‐treated mice was observed as early as at day 12 (*P* < 0.05). After 2 weeks of DSS withdrawal, there was 8% increase in body weight in Oxo‐treated mice compared to vehicle‐treated mice (Fig. 5B; *P* < 0.05). The microscopic progression of DSS‐induced colitis at day 19 includes an increase in the infiltration of inflammatory cells into the mucosa and submucosa (Fig. 5C). At day 19, partial regeneration of crypts and reepithelialization were observed; however, still there was a marked damage in colon mucosal architecture (Fig. 5C). Treatment with the vehicle had no beneficial effect on mucosal degeneration. In contrast, treatment with Oxo, especially at a dose of 5 mg/kg body weight, resulted in regeneration of crypts and reepithelialization (Fig. 5C). Also, infiltration of inflammatory cells in the colon mucosa and submucosa was prevented in Oxo‐treated mice (Fig. 5C).

In the recovery model of colitis, even after 2 weeks of DSS removal, there was 22% reduction in colon length (*P* = 0.0001) compared to the experimental group not treated with DSS (Fig. 5D). Treatment with Oxo resulted in 16% recovery (*P* = 0.0005) in the colon length of DSS‐exposed mice (Fig. 5D). Treatment with vehicle had no beneficial effect on DSS‐induced disease activity or histopathology in semichronic IBD (Fig. 5D). However, a significant reduction in disease activity (*P* = 0.001) (Fig. 5E) and histopathology scores (*P* = 0.001) were observed in Oxo‐treated mice compared to vehicle‐treated mice (Fig. 5F). Although less prominent than the acute form of DSS‐IBD, elevated levels of proinflammatory cytokines were also detected in the recovery phase (Fig. 5G). Treatment with Oxo significantly inhibited IL‐6 (*P* = 0.01), TNF‐*α* (*P* = 0.006) and IL‐12 (*P* = 0.02) expression in the colonic tissues compared to the vehicle‐treated groups (Fig. 5G). Phosphorylation of TAK1 at the recovery phase of DSS‐colitis was markedly reduced in Oxo‐treated mice to a level comparable to control mice treated with vehicle only (Fig. 5H).

It is notable that in both acute and recovery phase of DSS‐colitis, Oxo treatment facilitated epithelial monolayer integrity and restitution, most likely because of the profound decrease in mucosal proapoptotic cytokine expression. Together, our data suggest that activation of TAK1 is required for driving pathogenesis in DSS‐induced IBD.

### Inhibition of TAK1 reduces pathogenesis in NSAID‐induced IBD in IL‐10^−/−^ mice

**Figure 6:**
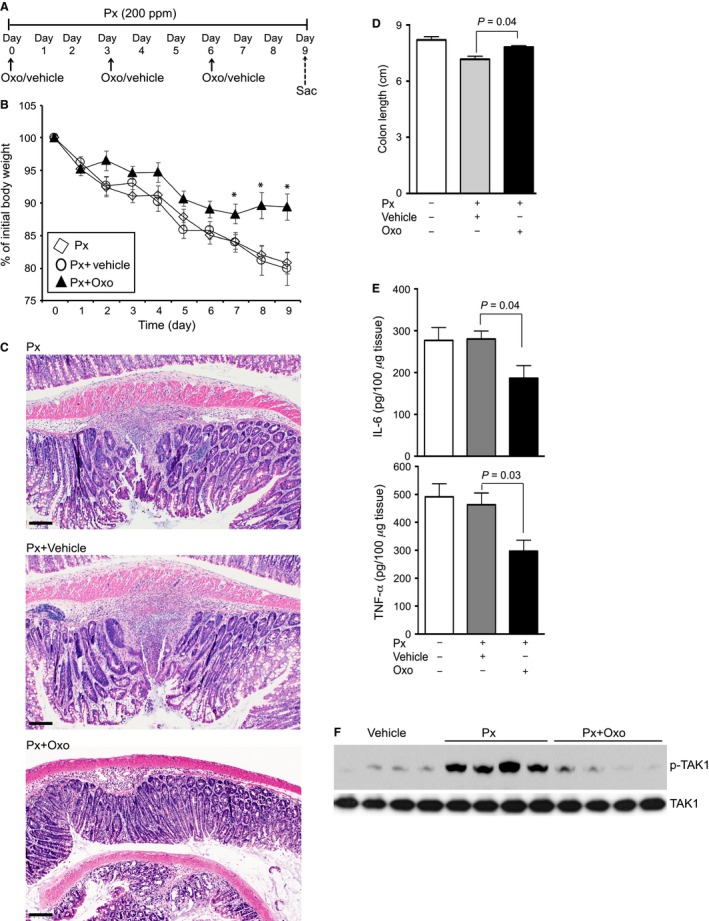
Inhibition of TAK1 reduces pathogenesis in NSAID‐induced IBD in IL‐10^−/−^ mice/. (A) 6‐ to 8‐week‐old male and female C57B/6 mice (*n* = 6/group) were on 200 ppm piroxicam (Px) and with vehicle (DMSO + mineral oil) or TAK1 inhibitor Oxo (5 mg/kg) and sacrificed as indicated. (B) Loss in body weight. Data presented as mean ± SEM. *P < 0.05 versus Px+vehicle. (C) H&E stained colons tissues; magnification: 200×; scale bar: 100 *μ*mol/L. (D) Colon lengths; (E) Expression of IL‐6 and TNF‐*α* in colon homogenates were analyzed by ELISA. Data presented as mean ± SEM.

NSAID (Piroxicam)‐accelerated IBD in IL‐10^−/−^ mice highly resembles human IBD in terms of heterogeneous pathogenesis and impaired epithelial integrity in disease development (Holgersen et al. 2014). To examine whether activation of TAK1 is a general mechanism in driving the pathogenesis, the role of TAK1 in NSAID/IL‐10^−/−^ model of IBD was assessed. Mice were treated with piroxicam (Px) and Oxo (5 mg/kg body weight) as indicated in Figure 6A. Untreated IL‐10^−/−^ mice at 6 weeks of age did not exhibit gross colonic inflammation; however, colitis was found in 100% of the IL‐10^−/−^ mice treated with Px (data not shown). Similar observations were reported by Berg et al. (2002). Treatment with Px resulted in gradual loss in body weight (Fig. 6B). Treatment with vehicle had no significant effect on Px‐induced loss in body weight in IL‐10^−/−^ mice. However, Oxo significantly prevented reduction in body weight (*P* < 0.05; Fig. 6B). After 9 days of Px treatment, moderate loss of crypts and multifocal inflammatory infiltrates were observed in the lamina propria, predominantly in the proximal colon. Epithelial hyperplasia and occasional ulceration were found at the inflammatory lesions. There was no detectable change in microscopic pathogenesis in Px‐exposed IL‐10^−/−^ mice treated with the vehicle. Notably, treatment with Oxo mitigated restricted infiltration of inflammatory cells and reduced thickening of the muscularis mucosa (Fig. 6C). The loss of crypts and epithelial damage were partially prevented in Oxo‐treated IL‐10^−/−^ mice. Also, the shortening of colon length in IL‐10^−/−^ mice were significantly prevented by Oxo treatment (Fig. 6D; *P* < 0.05). Moreover, elevated levels of IL‐6 and TNF‐*α* expression in IL‐10^−/−^ mice were significantly reduced by the treatment with Oxo but not vehicle (Fig. 6E; *P* < 0.05). Phosphorylation of TAK1 in Px‐exposed IL‐10^−/−^ mice was substantially prevented by the treatment with Oxo to a level comparable to control mice treated with vehicle only (Fig. 6F). Together, pharmacological inhibition of TAK1 activation was beneficial in different mouse models of IBD.

### Activation of TAK1 in F4/80^+^ macrophages in the recovery phase of DSS‐colitis

**Figure 7:**
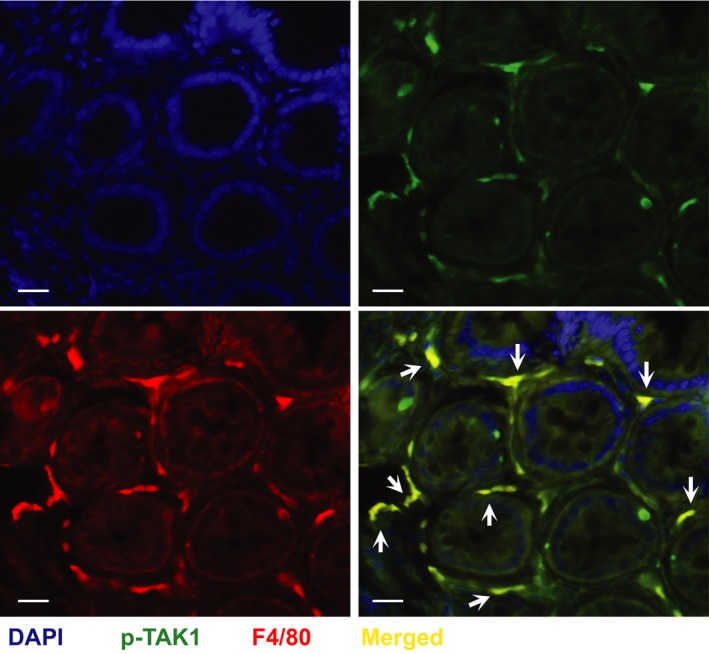
Abundance of phospho‐TAK1 in F4/80^+^ macrophages in the recovery phase of DSS‐colitis. Male and female C57BL/6 mice (*n* = 6–8/group) received 3% DSS or tap water for 5 days and thereafter only tap water for another 14 days and sacrificed on day 19. Representative IF images show phospho‐TAK1^+^ macrophages (yellow: merged) in colons. DAPI (blue), phospho‐TAK1 (green), F4/80 (red). Magnification: 200×; scale bar: 100 *μ*mol/L.

Although the immunofluorescent staining in Figure 2E demonstrated nonepithelial expression of activated TAK1 in IL‐10^−/‐^ model of colitis, the cell type(s) remains to be identified. Among nonepithelial cells, macrophages are considered as the primary responders in IBD. Infiltration of macrophages is elevated in colonic biopsy samples from IBD patients, and produce large amounts of proinflammatory cytokines, chemokines, and retain respiratory burst activity (Murch et al. 1993; Rugtveit et al. 1994, 1995, 1997; Smith et al. 2005; Smythies et al. 2005; Schenk et al. 2007; Kamada et al. 2008). We assessed TAK1 phosphorylation in F4/80^+^ macrophages in DSS‐induced semichronic IBD. TAK1^+^ cells (Green) in the colon mucosa were found to be predominantly F4/80^+^ cells (Red), as demonstrated by costaining (Fig. 7). Therefore, at the recovery/semichronic phase of DSS‐colitis, majority of TAK1^+^ cells in the colon mucosa were macrophages.

## Discussion

In this study, we define a new mechanism for the intestinal inflammation. We hypothesized that dysregulated activation of TAK1 facilitates colonic mucosal inflammation and subsequent pathogenesis in IBD. In support of this premise, we find hyper activation of TAK1 in patients with active UC or CD, as well as in murine models of IBD. We provide evidence for substantial reduction in IBD‐associated colon mucosal pathogenesis and clinical symptoms in experimental IBD by pharmacological inhibition of TAK1 activity. Further, we demonstrate that GC, a first line of treatment for IBD, fails to restrain activation of TAK1 and subsequent inflammatory reactions in mouse IBD.

The function of TAK1 in IBD is enigmatic and remains to be discerned. Several recent studies indicated association of TAK1 activation with the disease pathogenesis in IBD. In contrast, selective deletion of TAK1 in intestinal epithelial cells demonstrated cytoprotective roles of TAK1 in DSS‐colitis. In this study, we demonstrated upregulation in TAK1 phosphorylation in colonic tissue specimens from pediatric patients with UC or CD, indicating elevated TAK1 activation in IBD (Fig. 1). While there are differences between pediatric and adult IBD in terms of the clinical course, the etiology of the disease is thought to be similar (Kelsen and Baldassano 2008). Further, symptoms for pediatric IBD are similar to those observed in adults (McMullin et al. 2014). We anticipate that a similar profile for TAK1 activation would be observed in adult IBD patients. However, additional studies are required to address this. Nevertheless, we found that signaling intermediates downstream of TAK1 activation, such as NF*κ*B, JNK, and p38 MAPK, were also activated in pediatric IBD patients (Fig. 1). These observations were further supported by the upregulation of TAK1, NF*κ*B, JNK, and p38 MAPK activation in two distinct murine models of IBD (Fig. 2). Together, we find activation of TAK1 as an integral part of human and murine IBD. These results are concordant with a recent study that suggested positive correlation between RKIP (a kinase upstream to TAK1) activation and disease severity in IBD patients, as well as between RKIP and TAK1 phosphorylation in DSS and TNBS models of colitis (Lin et al. 2016). Our immunofluorescence studies indicated elevated p‐TAK1 expression, prominently in nonepithelial cells (Fig. 2 E and F). Here, we used epithelial injury/repair model (DSS‐colitis) and epithelial injury/adaptive immune hyperactive model (NSAID/IL‐10^−/−^) of human IBD, which exhibit predominant proinflammatory roles and involve innate immune cells including neutrophils and macrophages. It is plausible that during intestinal inflammation, TAK1 exerts cell‐specific distinct functions. For example, TAK1 in intestinal epithelial cells maintains mucosal homeostasis, while TAK1 in innate immune cells promotes mucosal inflammation and subsequent pathogenesis. In support of this premise, MyD88, a TLR‐adapter protein pivotal for TAK1 activation, has pathogenic and protective roles in leukocytes and epithelial cells, respectively, in mouse IBD (Jobin 2010; Muhlbauer et al. 2013). Beneficial and detrimental roles of NF*κ*B in intestinal homeostasis and diseases have been demonstrated previously (Karrasch and Jobin 2008). Moreover, TNF, a TAK1‐activating ligand, has both protective and pathophysiological roles in IBD (Edelblum et al. 2008; Dube et al. 2015; Punit et al. 2015).

To date, there is no direct evidence for functional roles of TAK1 in facilitating inflammation and associated pathogenesis in IBD. Here, we performed a comprehensive study to determine whether TAK1 is dispensable in coordinating colon mucosal inflammation. To examine the therapeutic benefit of TAK1 inactivation, mice were treated with Oxo, a highly selective pharmacological inhibitor of TAK1 following the exposure to DSS. At first, we investigated the effects of Oxo on DSS‐induced acute IBD. Treatment with Oxo at a dose of 1 or 5 mg/kg body weight alleviated the clinical symptoms and micro/macroscopic pathogenesis in acute IBD (Fig. 3). Consistently, Oxo restrained activation of TAK1 and the downstream signaling intermediates that contribute to inflammatory reactions including synthesis of proinflammatory cytokines (Fig. 4). These results suggest that the suppression of TAK1 activation ameliorate pathogenesis in DSS‐induced acute IBD. However, at a dose of 10 mg/kg body weight or higher, Oxo exhibited some toxicity in DSS‐treated mice. Since TAK1 is required for several cellular functions, it is plausible that complete inhibition of TAK1 activity at relatively high doses of Oxo might have interfered with physiological homeostasis and thereby introduced toxicity. Nevertheless, inhibition of TAK1 activity was indeed beneficial in acute IBD.

A limitation of experimental IBD is the unavailability of a single‐animal model that represents all major characteristics of human IBD. IL‐10^−/−^ mice develop IBD spontaneously. The disease phenotypes and histopathology in IL‐10^−/−^ mice are grossly different from DSS‐colitis, especially for the heterogeneous intestinal pathogenesis that resembles CD (Holgersen et al. 2014). To examine the generality of TAK1 activation as an important mechanism for IBD, we investigated the effect of TAK1 inhibition on colon mucosal pathogenesis in IL‐10^−/−^ mice. To synchronize the onset and accelerate the development of IBD, IL‐10^−/−^ mice were administered piroxicam/NSAID in chow (Berg et al. 2002). Like DSS‐induced acute IBD, pharmacological inhibition of TAK1 exhibited profound recovery in IL‐10^−/−^ mice from piroxicam‐mediated colon mucosal pathogenesis. These results implicated elevated activation of TAK1 as a common mechanism for intestinal inflammation.

Since IBD is a chronic inflammatory condition of the gastrointestinal tract, we extended our studies to investigate the roles of TAK1 in the recovery phase of DSS‐induced IBD. This model was established by Melgar et al. (2005) and later successfully reproduced by several independent studies (Sha et al. 2013). At the recovery phase of the disease, we observed moderate‐to‐low activation of TAK1, JNK, and p38 MAPK compared to acute DSS‐induced IBD (Fig. 2B). Also, the expression levels of TNF‐*α* and IL‐6, the hallmarks of intestinal inflammation were lower in the recovery phase compared to acute IBD (Figs. 4B and 5G). Nonetheless, activation of TAK1 and the downstream signaling mediators as well as the proinflammatory signatures were still substantially higher than the control/untreated mice. Consistent with these observations, persistent loss in body weight, disease activity, shortening in colon length, and microscopic histopathology were found till the end‐point (19 days) of the study, while DSS was withdrawn after 5 days of treatment (Fig. 5). In the recovery model of IBD, we treated mice with Oxo after 4 days of DSS withdrawal, the transition period from acute to semichronic IBD. Pharmacological inhibition of TAK1 prevented most of the above pathogenesis in the recovery phase, at least in part. However, the beneficial effects of Oxo in maintaining colon mucosal architecture and restraining inflammatory infiltrates were more pronounced in the acute phase compared to recovery phase of the disease. It is likely that TAK1 activation, which promotes intestinal inflammation predominantly occurs at the acute stage. However, the residual TAK1 activation that persisted in the recovery phase was also suppressed by Oxo. Hence, inhibition of TAK1 activity was beneficial for the recovery phase as well as the acute stage of the disease. Our data suggest uncontrolled activation of TAK1 may account for the inflammation‐related pathologies in IBD. GC therapies are the standard treatment for moderate‐to‐severe IBD as they facilitate rapid remission of disease activity. However, there was no additional beneficial effect from GC treatment while mice were treated with both Oxo and Dex (Fig. 3).

Notably, we find suppression of TAK1 activation is beneficial in colitis, while previous reports suggest protective roles of epithelial TAK1 (Kajino‐Sakamoto et al. 2008; Kim et al. 2009). Cell‐specific roles of signaling mediators and inflammatory agents in inflammatory bowel disease are an emerging field of research. MyD88, a TLR‐adapter protein pivotal for TAK1 activation, has pathogenic and protective roles in leukocytes and epithelial cells, respectively, in mouse IBD (Jobin 2010; Muhlbauer et al. 2013). Moreover, TNF, a TAK1 activating ligand, has both protective and pathophysiological roles in IBD (Edelblum et al. 2008; Dube et al. 2015; Punit et al. 2015). Intriguingly, at the early stage of the disease (after 3 days of DSS treatment), the activation of TAK1 is restricted in the epithelial cells, while TAK1 activation in the nonepithelial cells is negligible. We posit that epithelial TAK1 contributes less at the advanced stage of the disease and Oxo‐dependent suppression of colonic inflammation is primarily mediated via inhibition of TAK1 activation in the nonepithelial immune cells in the lamina propria. In support of this premise, costaining studies suggest that TAK1 phosphorylation predominantly occurs in macrophages (Fig. 7). Additional studies involving mice with conditional deletion of TAK1 in macrophages are required to ascertain the roles of macrophage‐derived TAK1 in IBD.

Collectively, we demonstrate activation of TAK1 as a critical mechanism for colon mucosal inflammation. Targeting inflammation‐inducing cell signaling mediators has been an active area for new drug discovery in IBD. TAK1 is upstream of many inflammatory pathways, such as MAPK and JAK‐STAT, currently targeted in IBD (Van Den Blink et al. 2002; Bamias et al. 2016). Therefore, a major consequence of targeting TAK1 would be higher anti‐inflammatory efficacy by concomitant suppression of many pathways. One attractive mechanism, then, by which inflammatory outcomes in IBD could be prevented is through pharmacological inhibition of TAK1 action. A limitation of this approach would be the collateral suppression of TAK1 activity in intestinal epithelial cells that is required for colon mucosal homeostasis. Future studies on mucosal cell‐specific TAK1 activation would be a prelude for cell‐restricted TAK1 inhibition as an alternative strategy to treat IBD.

## Conflict of Interest

No conflicts of interest, financial or otherwise, are declared by the authors.
